# Toward an Asian-based bodily movement database for emotional communication

**DOI:** 10.3758/s13428-024-02558-2

**Published:** 2024-12-10

**Authors:** Miao Cheng, Chia-huei Tseng, Ken Fujiwara, Shoi Higashiyama, Abby Weng, Yoshifumi Kitamura

**Affiliations:** 1 Research Institute of Electrical Communication, Tohoku University, Sendai, Japan; 2 Interdiscipinary ICT Research Center for Cyber and Real Spaces, Tohoku University, Sendai, Japan; 3 Department of Psychology, National Chung Cheng University, Chiayi, Taiwan; 4 Graduate School of Information Sciences, Tohoku University, Sendai, Japan; 5 Said Business School, University of Oxford, Oxford, UK

**Keywords:** Emotion, Body motion, Database, Asian

## Abstract

Most current databases for bodily emotion expression are created in Western countries, resulting in culturally skewed representations. To address the obvious risk this bias poses to academic comprehension, we attempted to expand the current repertoire of human bodily emotions by recruiting Asian professional performers to wear whole-body suits with 57 retroreflective markers attached to major joints and body segments, and express seven basic emotions with whole-body movements in a motion-capture lab. For each emotion, actors performed three self-created scenarios that covered a broad range of real-life events to elicit the target emotion within 2–5 seconds. Subsequently, a separate group of participants was invited to judge the perceived emotional category from the extracted biological motions (point-light displays with 18 or 57 markers). The results demonstrated that the emotion discrimination accuracy was comparable to Western databases containing standardized performance scenarios. The results provide a significant step toward establishing a database using a novel emotional induction approach based on personalized scenarios. This database will contribute to a more comprehensive understanding of emotional expression across diverse contexts.

**Supplementary information:**

The online version contains supplementary material available at 10.3758/s13428-024-02558-2.

## Introduction

The role of bodily movements in emotional communication has been recognized since the time of Darwin (1872/1998), although the number of studies is significantly smaller than those on facial emotion (Witkower & Tracy, 2019). The trend (or even mandate) to wear face masks, which became widespread during the COVID-19 pandemic, highlighted the importance of affective cues from bodies when faces are not visible.

### Role of body movements in the expression of emotions

According to Planalp et al. (2006), emotions are the result of the cognitive interpretation of both physiological and behavioral reactions to stimuli. This definition underscores a crucial aspect for researchers striving to decipher the complexity of human emotions; that is, while emotional experiences are internal, they have the power to influence human behavior and, thus, the external world—particularly interpersonal interactions. Individuals reveal their internal worlds through speech, facial expressions, and bodily gestures; at the same time, they attempt to understand others by trying to decode as much as possible of the external signals received. While researchers have been fully aware of the importance of the expression and perception of emotions, there is still much we do not know. In particular, earlier research focused mostly on verbal cues and facial expressions, leaving the realm of bodily emotions largely unexplored. Several studies using both static and dynamic whole-body stimuli have demonstrated that people recognize emotions conveyed through body language as accurately as they do through facial expressions (Hadjikhani & de Gelder, 2003; Atkinson et al., 2004), highlighting the need for further research in this area.

In fact, de Gelder (2009) suggested that using bodily expression stimuli in the study of emotions can offer an even broader perspective on emotional signals. There is, for instance, a clear advantage held by bodily emotions over facial emotions when communication takes place over a greater physical distance. Furthermore, reading emotional cues from the body rather than the face could potentially eliminate the additional distraction of facial features and, therefore, personal identity. The author also noted that for those with visual impairments, facial expressions may be hard to discern while bodily expressions could still be relatively easy to perceive. Finally, de Gelder stressed that in the real-world context, people comprehend facial expressions in conjunction with bodily expressions, frequently checking whether facial expressions are congruent with gestures. Aviezer and colleagues (2012) noted that emotion perceived from the face varied systematically with contextual body cues. In short, the movements of the body impact our ultimate judgment of communication (Meeren et al., 2005).

Understanding how the body expresses emotions is, therefore, crucial for social interactions which play an important role in virtually every field. Bodily emotion databases can provide researchers with robust data for further investigation in areas such as computing, education, medicine, and psychology. The research goal of this study is to develop a motion database for Asian bodily emotion expression, covering the complexity of emotion expression in the real world. We introduce a novel emotion induction method using personalized scenarios to capture a wide range of emotional expressions under various contexts. We also evaluate the recognition accuracy of this method. By focusing on Asian performers, this database addresses a gap in the field regarding underrepresented groups in emotion and body expression research. This paper provides the foundation for a comprehensive dataset that supports research across multiple fields, including emotion, behavioral, and cognitive science, as well as applications in affective computing.

### Cultural differences in bodily emotion expression

Many psychological phenomena have shown cross-cultural differences (Markus & Kitayama, 1991), and numerous studies have revealed considerable cross-cultural variations in behavioral patterns of emotional expressions (e.g., Cordaro et al., 2018; Ekman, 1971; Jack et al., 2012; Jack et al., 2016; Matsumoto, 1990), including the body. These studies acknowledge the importance of adding cultural dimensions to embodied agents, as the interpretation of emotion is clearly shaped by culture. For example, Hall et al. (1996) reported that elementary school students from the USA and mainland China had significant differences in their perception of posture and gesture communication, but not in facial expressions. Sogon and Masutani (1989) reported that Japanese participants were superior to American participants in detecting contempt and surprise from bodily-expressed emotions, but demonstrated no difference in other emotions. Scherer et al. (1988) reported that the Japanese use fewer hand, arm, and whole-body gestures than Americans when in emotional situations. Parkinson et al. (2017) investigated how participants from a remote village in Cambodia (an Asian country) recognized Western point-light displays of various emotions and reported that culturally specific cues were critical in deciphering emotional communication from a distant culture. Kleinsmith et al. (2006) used a motion-capture system to gather data from 13 human subjects and asked them to express various emotions through postures. The resulting data were then evaluated by observers from three cultures. Japanese observers performed slightly better in recognizing fear and anger postures than observers from the USA and Sri Lanka. This performance advantage may have been because the tested posture stimuli were mainly by Japanese performers, suggesting a possible own-race advantage to be further investigated. The result led the researchers to confirm that the introduction of additional cultural diversity can help in creating more believable and relatable, culturally aware avatars, ultimately enhancing the user experience in computing. Considering the comprehensive understanding of bodily emotion expressions from the cross-cultural psychology perspective as well as the practical benefit of improving interhuman relationships and even human–machine interactions, the disproportionate representation in major emotional databases may negatively influence future research.

### Existing bodily emotional expression databases and their limitations

Over the past few decades, many databases of dynamic body emotion expressions have made key contributions to advancing our systematic understanding of emotion recognition based on dynamic movements (for a summary, see Volkova et al., 2014a). Among the databases, the most common formats fell into two categories: video recordings (e.g., Wallbott, 1998; FABO, Gunes & Piccardi, 2006; Body Language Dataset, BoLD, Luo et al., 2020; GEMEP, Bänziger et al., 2006; iMiGUE, Liu et al., 2021; Spontaneous Micro-Gesture dataset [SMG], Chen et al., 2023) and motion-capture recordings (e.g., MPI, CMU Graphics Lab, KUG, USC CreativeIT database, Emilya, Communicative Interaction Database). The former are relatively low-cost compared to the latter, which require a system of multiple optical cameras to provide three-dimensional (3D) information (e.g., volume of the body, movement in depth). For applications that require high-precision 3D spatial information, such as the establishment of an automatic recognition system for affective states or animation/graphic generation, high-quality motion-capture recordings are invaluable.

Given that the exploration of bodily emotion expressions is still in its infancy, numerous knowledge gaps remain to be addressed. Most notably, we have noticed a glaring lack of cultural diversity. While efforts have been initiated to introduce more cultural inclusivity to linguistic and facial emotion databases such as the addition of Indonesian and Korean datasets (Saputri et al., 2018; Khanh et al., 2021), to the best of our knowledge, the same degree of effort has not been observed in the realm of research on bodily emotion expressions. For instance, Atkinson et al. (2004) considered the effect of intensity on emotional delivery, but all ten participants responsible for encoding the bodily emotions were students from a college in England (if they came from different cultural backgrounds, that factor was not explicitly stated or considered in the paper). More recent initiatives, such as the Berkeley Multimodal Human Action Database (MHAD; Ofli et al., 2013), the Emotional Body Database (EBD; Dael et al., 2012), the Geneva Multimodal Emotion Portrayals Corpus (GEMEP) offered by the Swiss Center for Affective Sciences (Bänziger et al., 2012), and Emilya: Emotional body expression in daily actions database (Fourati & Pelachaud, 2014), primarily consist of samples from Western individuals. This oversight echoes the concerns raised by other researchers, who have criticized the behavioral sciences for their overreliance on Western populations, often neglecting broader cultural perspectives that are essential for a comprehensive understanding of human emotions (Arnett, 2009; Henrich et al., 2010).

In a rare case where the research database does recognize the importance of including cultural diversity, such as in the MPI (Max Planck Institute) Emotional Body Expressions Database (Volkova et al., 2014a), there is still much room for improvement, as the specific cultural background of the eight actors remains unclear, and the researchers only include information in their native tongues. Furthermore, in total, there are only three different native tongues spoken by the participants, two of which are Western languages (German and English); the only non-Western variation is Hindi, and there is no information about the specific number of people who fall under this category, nor is there any mention of the possibility of bilingualism, which must be considered since high proficiency in English is a requirement for recruitment in this case.

Another limitation found in most bodily movement databases is the induction of emotions from limited scenarios provided by professional directors or experimenters (e.g., GEMEP, MPI). This common practice, possibly based on the assumption that emotion is universal, has recently been challenged by studies that suggest our culture and background do modulate the way we experience emotions (Jackson et al., 2019; Kitayama et al., 2000; Mesquita & Frijda, 1992). In other words, people from different cultures may not feel the same emotion in a given scenario. To provide more personalized and culturally sensitive emotion stimulation, we proposed a novel approach using personalized scenarios that were not specified by others but created by the performers themselves for each emotion performed. This approach increases (1) diversity and (2) personalization of emotion induction cues, and reflects the individual and cultural nuances of emotions in Japan. By incorporating the unique cultural context and expertise of Japanese performers, we aim to provide a more comprehensive understanding of the interplay between emotions, cultural context, and body movements. As artificial intelligence rapidly advances, our effort directly supports the increasing demand for more diverse and fair representation in training data to avoid perpetuating inequality on a global scale (Zou & Schiebinger, 2018).

### Current study

Given the importance of inclusivity and diversity in our collective knowledge, we are committed to expanding the current repertoire of human bodily emotions by introducing a new database that includes Asian participants, thereby broadening the range of existing data available to the research community. Specifically, we chose to begin with Japan, as it is a region in Asia that has offered many research findings on cross-cultural emotion expression (e.g., Matsumoto, 1990). Several considerations influenced the choice of Asian (Japanese) participants. First, as mentioned above, the Asian population is one of the most underrepresented populations in terms of body emotion studies. Secondly, Asian cultures represent a large group of distinct cultures, yet also share some similarities according to cultural dimensions (Hofstede, 2003). Japanese participants are frequently recruited in cross-cultural comparison studies due to the development of their research culture and history. Finally, setting up a motion-capture lab requires resources (e.g., budget, space, personnel), and we started from where these resources were conveniently available.

In this study, performers were asked to express eight emotions—seven basic emotions (joy, anger, sadness, fear, contempt, disgust, surprise) based on Ekman (1992) and a neutral state—using whole-body movement. These emotions were selected not only because they were considered basic and universal (Ekman & Friesen, 1986; Ekman, 1992; Matsumoto, 1992; Levenson, 2011), but also because they were the most commonly included emotions in existing databases, providing a good benchmark and comparison basis. We used both a video camera and a motion-capture system for data collection but focused on the 3D information from the latter because of its wider potential in future applications. Additionally, it has the advantage of presenting the full-body motion in minimalistic and dynamic point-light displays (PLDs; Johansson, 1973), free from factors unrelated to motion (e.g., likability of the performing agent or environmental contexts), yet sufficient to trigger social-cognitive perception conveyed from the movements (Hirai & Senju, 2020).

To evaluate the Japanese performers’ bodily expressions, we invited additional participants to judge the perceived emotions from the PLDs derived from the performance recordings. Because our recordings include different emotional scenarios by each performer, we expect more noise and a lower accuracy rate than those from fixed performance scripts. To focus on our research interest in bodily movements, we reduced our movement recordings to point-light displays (i.e., biological motion; Johansson, 1973) to remove the influences from the form aspects. This enabled us to also relate our studies to other biological emotion databases (e.g., Dittrich et al., 1996; Atkinson et al., 2004) and databases consisting of only stick figures (e.g., Volkova et al., 2014a). Our motion-capture system provides a full-body skeleton captured by 57 markers, whereas most previous studies on emotional recognition accuracy of body motion with a skeleton (e.g., biological motion) were based on fewer markers (e.g., Dittrich et al., 1996; Atkinson et al., 2004). To relate our study to the previous findings and make our results comparable with them, we further reduced the number of markers to 18 to create a partial condition. With both partial and full conditions, we will also be able to investigate the effects of marker numbers on body emotional recognition.

In short, we present a pioneering emotion-motion database that utilizes a novel emotion-triggering paradigm, meticulously designed to extend the cultural scope of current resources. Our primary aim is to foster a more balanced representation in the study of emotions.

## Method

### Bodily emotion database collection

#### Target emotions

Seven universal emotions defined by Ekman and Friesen (1986) were selected for the current study: joy, anger, sadness, fear, contempt, disgust, and surprise. We also included a neutral emotion as a point of baseline comparison. The Japanese and English definitions of the emotions are included in Supplementary Table 1.

#### Performers

Six Japanese professional performers (two male and four female) from a professional theater group, ranging in age from 21 to 63 years, with a mean age of 31 years and mean performance experience of 17 years, were recruited. They came to the motion-capture lab at Tohoku University and performed the eight target emotions (joy, anger, sadness, fear, contempt, disgust, surprise, and neutral). Each performer prepared their own performance scenarios prior to the filming day. No actor saw any others’ performances prior to the study session, ensuring that each actor's performance remained uninfluenced by others, thereby allowing for a wide range of interpretations and patterns of movements. They received monetary compensation at a professional rate for their time.

#### Performance scenario

Each performer prepared three different scenarios for each target emotion, yielding a total of 3 × 6 = 18 scenarios. For example, for joy, performer 5’s three scenarios are as follows:

- When I am having a meal
- Reunion with friends
- Winning a lottery

The performers are all native Japanese, so they submitted the performance scenario list in the Japanese language. Supplementary Table 2 includes the translated scenarios from all six participating performers 1–6. Although the submitted lists appear simple, most performers told us during the interviews that they had more detailed plans in mind (e.g., factors of consideration including their social roles in the scenarios and the details of their environmental contexts) determined in advance. Therefore, we conducted an interview at the end of the experiment to obtain more details regarding these choices.

During data acquisition, we asked the performers to express all the emotional scenarios (except neutral) with three intensities: low-, mid-, and high-intensity. For the neutral state, they only performed at one intensity level. In total, each performer recorded 7 emotions × 3 scenarios × 3 intensities + Neutral × 3 scenarios × 1 intensity = 66 performances. Each performance lasted 3–5 seconds.

#### Motion capture

The motion-capture data acquisition was completed in the Research Institute of Electrical Communication at Tohoku University with the Vicon motion-capture system (Vicon Motion Systems Ltd., UK) and Vicon Shogun Live 1.7 software. The system has 12 optical cameras (Vicon Vero X), with a recording area of 4.7 m × 5.8 m.

Upon arrival, actors followed the COVID-19 infection control measures, including measuring body temperature, using hand sanitizer, and wearing face masks. Actors then received instructions on the data collection and signed the informed consent form.

**Fig. 1:**
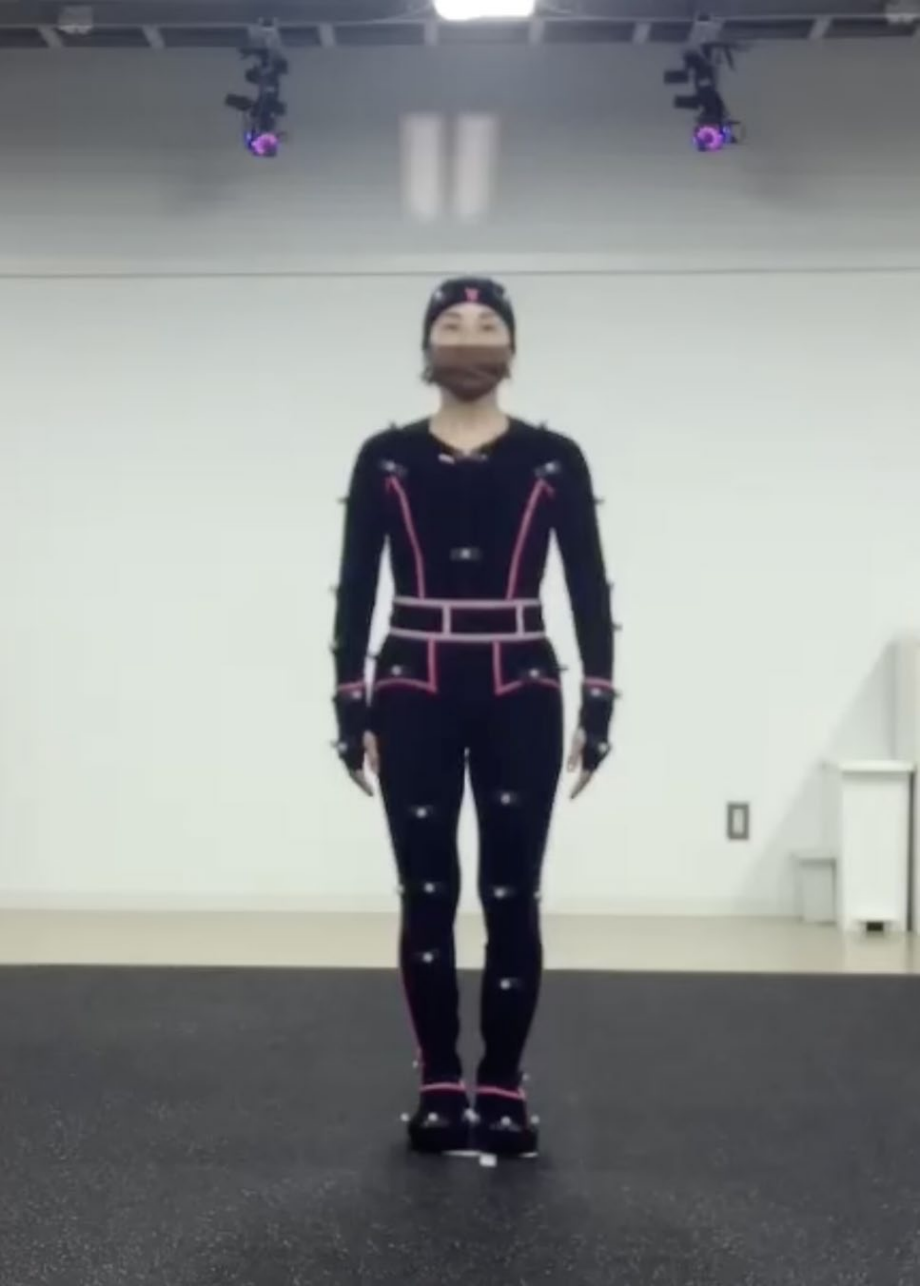
A professional performer in a motion-capture suit with 57 markers attached to various joints and body segments

In their tightly fitted Vicon whole-body suits (including a cap, gloves, shoes, and top and bottom suits), the actors had 57 retroreflective markers attached to various joints and body segments (Fig. 1). The skeleton model for each participant was created using the marker set “Production,” which included 57 markers. Before the formal body movement recording, the actors practiced and familiarized themselves with moving their bodies in the suits. The actors were directed to perform the emotional postures in their own way; thus, no constraints were placed on them.

For each emotion, the actors performed a portrayal for 3–5 seconds using whole-body movement. Between the scenarios, they were allowed to take breaks to adjust their emotional status for the next portrayal. Each performance was recorded with a video camera from the front. After each body movement recording, the actors were interviewed about the scenario.

**Fig. 2:**
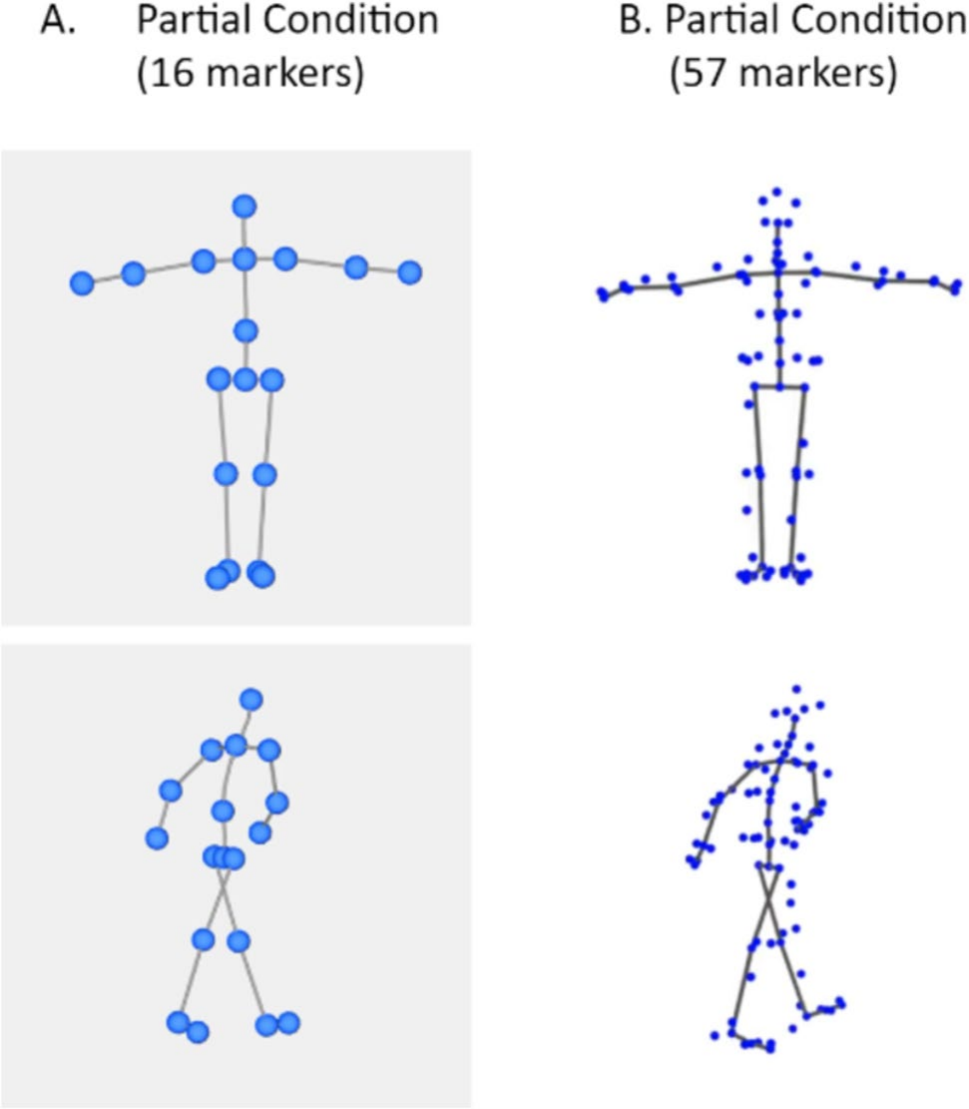
Examples of **A** partial and **B** full conditions

Using the Vicon Shogun Post 1.7 software, the motion-capture data were adjusted to face front view and exported to “.bvh” (for partial condition) and “.fbx” (for full condition) files. Then, we used MATLAB R2022a and motion style toolbox (Förger & Takala, 2015) to convert the .bvh files into point-like biological motion videos with 16 markers (partial condition) and Blender 3.1.0 (Community, B. O., 2018) to convert .fbx files into biological motion videos with 57 markers (full condition) (Fig. 2).

#### Performance interview

After all the motion-capture sessions were completed for each performer, we asked them to rest before an interview session, which typically took less than 1 hour. The purpose of the interview session was twofold. First, the performer had a chance to watch all 66 of their performance recordings on a PC together with the experimenter. If the performer was not satisfied, they had the option for a retake, in which case the new recording would be used to replace the old one. The request for a retake was rare, however, occurring with only one performer in one trial. Secondly, we allowed the performers to elaborate on their performance scenarios and acting strategies. This was particularly helpful in cases when the simplified list was not intuitive to us at first sight (as the list was used as a reminder for the performers’ acts). In addition, the performers also shared how they utilized their body motions to express their intended emotional effects. This interview session followed a semi-structured format, and some performers noted the difficulties they experienced during the process. On average, the interview session lasted between 30 and 60 minutes.

The interview was recorded with a voice recorder, and we referred to it when we constructed and translated the scenario list and the contextual analysis.

### Evaluation: Bodily emotion recognition test

#### Overview

This task required participants to watch a short clip on our chosen online experiment platform, Labvanced (www.labvanced.com), and judge which emotion was perceived at the end of the video. If needed, the participants could replay the video until they felt confident in selecting one answer from a predetermined list of eight choices: joy, anger, sadness, fear, contempt, disgust, surprise, and neutral. The participants were required to finish watching the whole clip before jumping to the next trial, and the order of the eight choices was randomized in each trial to avoid any response bias. Each participant received a unique experiment link to access the online experiment.

#### Participants

Twenty-five university students from Asian countries (Hong Kong, Japan, and Taiwan) participated in this study (19 female and 6 male, average age = 20 years). The sample size was determined based on a similar study by Volkova and colleagues (2014b). They reported average accuracy of 35.2% from 32 participants with an effect size (*η*_*p*_^2^) of 0.28. Therefore, with an estimated *η*_*p*_^2^ of 0.28, we expected an effect size *f* of 0.62. Then the power analysis was conducted using G*Power (Faul et al., 2007), yielding a minimum sample size of 10 needed for an *F*-test (repeated-measures within-factor analysis of variance [ANOVA]) to reach a power of 0.90 with alpha at 0.05. Combining the power analysis and sample size of previous studies, the appropriate number of participants was between 10 and 32. In the end, we recruited 25 participants to allow a balanced presentation of the stimulus order sequence.

Because the experiment was conducted on an online platform during the pandemic, we recruited participants from the university network (including students, staff members, and their connections) as this was the group with reliable access to infrastructure resources (e.g., internet, computers) and better technological knowledge (e.g., navigating online platforms). Participants received a briefing via a video conference (Zoom) before giving their written informed consent. They received either 2000 Japanese yen or 400 New Taiwan dollars for their time. The University Human Research Ethics Committee approved all the experimental procedures. All methods were performed in accordance with the relevant guidelines and regulations.

#### Stimuli and procedures

With this being an online experiment, our participants could choose the time and location to complete the experiment. As this study was conducted at the beginning of the pandemic, at times when participants were unfamiliar with the online experiment platform, we scheduled individual appointments with them to explain the experimental procedures via a video conference (Zoom). During each appointment session, we first invited the participant to join a demo experiment with their own PC to ensure that they understood the workflow correctly. We then tested their internet and PC processor speed to ensure that their equipment met the requirements for the online experiment. The viewing details (e.g., distance, lighting) varied across participants because participants joined online from their chosen locations and devices, but they were asked to use the same device in the same location throughout all experimental conditions. The participants were instructed to adjust the viewing distance so that they could see the centered stimuli clearly, and the displays during the experiment automatically covered the participants’ whole monitor screen (i.e., full-screen mode). To minimize unwanted disturbances from the environment, participants were instructed to join this experiment from a quiet place. We used the native language of our participants (English, Mandarin, or Japanese) to ensure accurate communication. The online platform instruction pages were multilingual and were proofread by our multilingual research team members; participants chose their preferred language to proceed.

The stimuli were the mid-level-intensity bodily emotion videos from the six performers mentioned above. We did not include videos of all levels here, as our primary interest in the current study was not the influence of emotional intensity, but whether bodily emotion videos from diverse scenarios were as recognizable as those from standardized scenarios. Our experiment employed a within-subject design. In total, each participant watched 8 emotions × 3 scenarios × 6 performers × 1 intensity (mid-level) × 2 conditions (partial and full markers) = 288 videos, separated into four blocks. Two blocks contained a partial marker condition, and two blocks contained a full marker condition. Each block contained videos from three performers, with a randomized sequence of emotions, lasting about 20–30 minutes. In total, it took 1.5 to 2 hours to complete the experiment including breaks. Participants were instructed to take additional breaks when needed. We randomized the sequence of testing trials/blocks for every subject to avoid any systematic bias from fatigue or testing order.

**Fig. 3:**
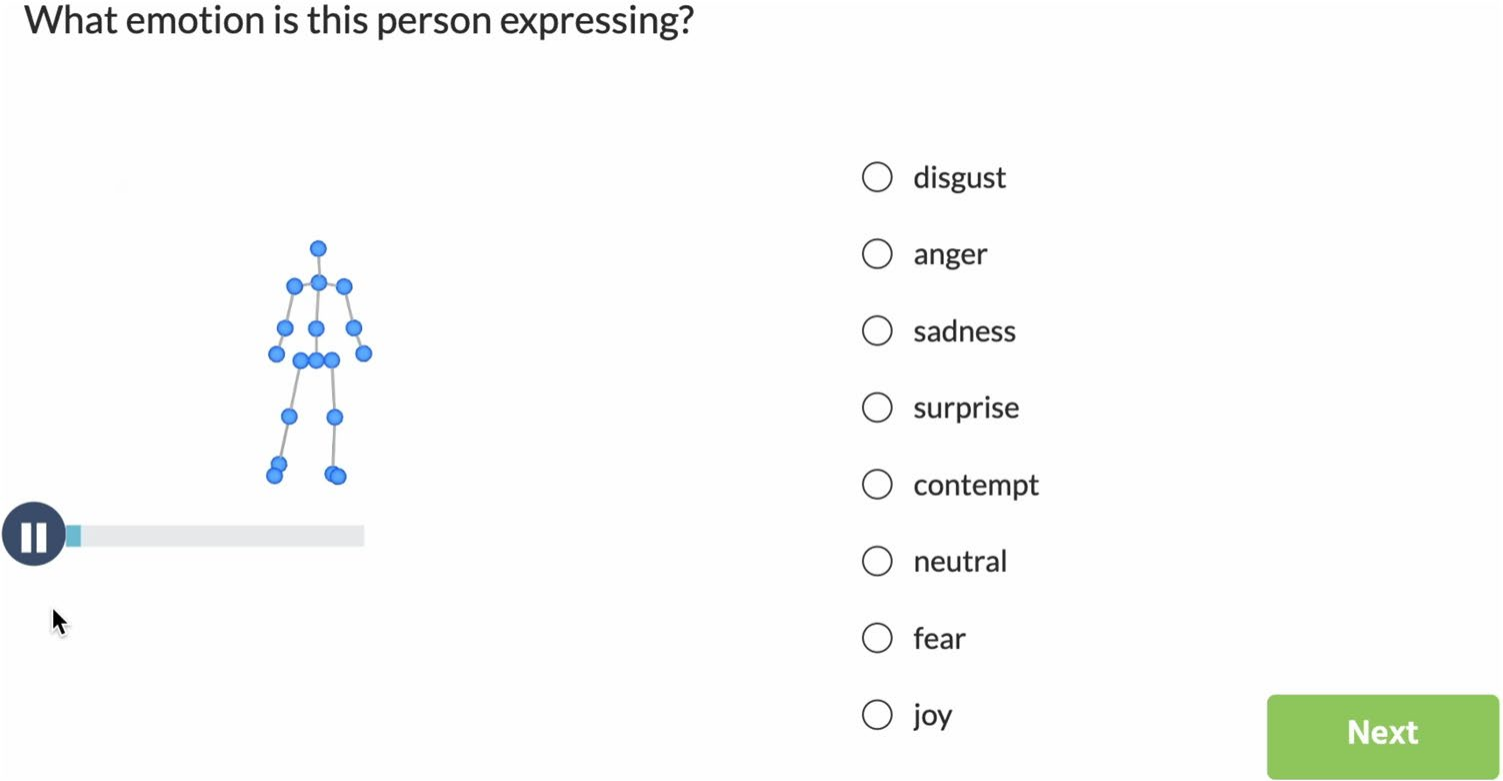
A sample trial. Participants viewed a short video (3–5 seconds) and selected an emotion that best described the video

Figure 3 is a sample screenshot of the experiment webpage. There was a break every 24 trials (i.e., three breaks in each session), and the participants could take additional breaks if they felt the need to.

## Results

Using the data collected, we developed a preliminary bodily emotion database comprising 792 biological motion videos (66 clips of performance × 6 performers × 2 marker conditions). For the evaluation experiment, 25 participants engaged in the emotion recognition task to evaluate the emotion expressions. The final data analysis was conducted on the responses of 22 participants, as we excluded three participants who did not complete the experiment. We calculated the intraclass correlation coefficient (ICC) to assess the interparticipant reliability of accuracy judgments across 22 participants in 16 conditions (8 emotions × 2 marker conditions). The ICC based on the ICC (3,k) model was computed using JASP, yielding a point estimate = 0.978 (95% CI [.958, .991]), indicating excellent agreement among the accuracy judgments (Koo & Li, 2016). To analyze the influence of gender on observers’ emotion detection ability (16 F, 6 M), we applied two-way ANOVA with gender (between-subject) and emotion (within-subject) on emotion recognition accuracy. Results showed no significant effect of gender, *F*(1,20) = 0.438, *p* = 0.562, *η*_*p*_^2^ = 0.017, or interaction effect between gender and emotion, *F*(7, 140) = 0.916, *p* = 0.496, *η*_*p*_^2^ = 0.044.

### Bodily emotion recognition accuracy across different performers

**Fig. 4:**
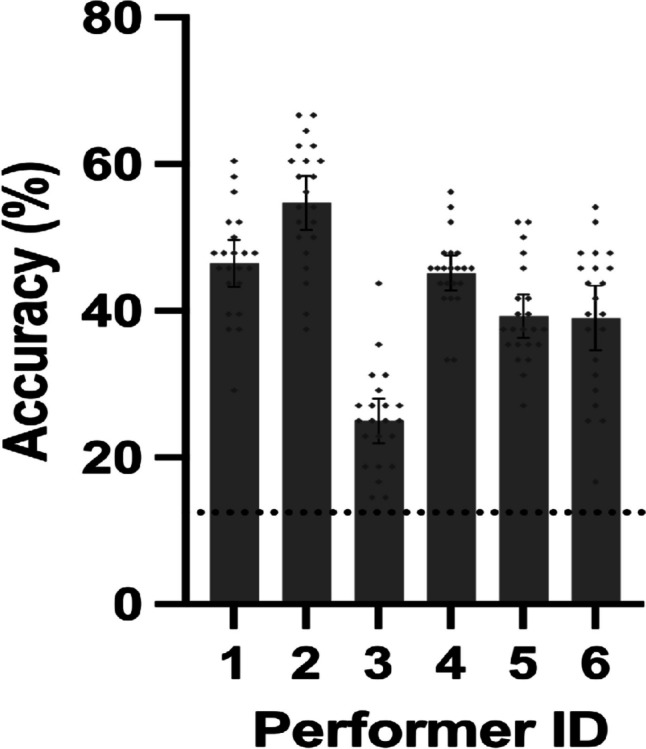
Mean and 95% CI of recognition accuracy for all performers

**Fig. 5:**
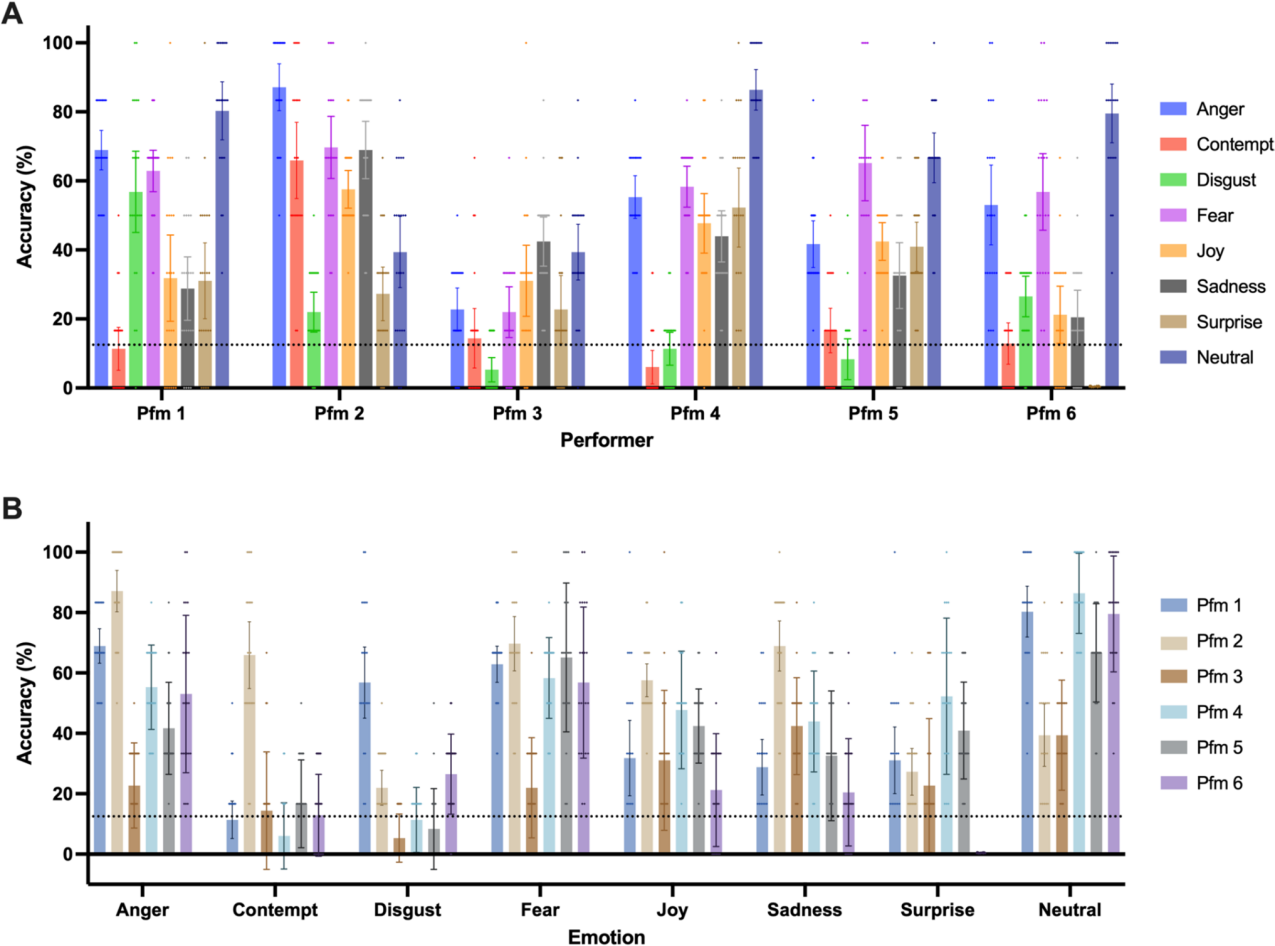
Mean and 95% CI of recognition accuracy of each emotion for all performers. For easy comparison, accuracy data are organized in two ways: **A** grouped by performer and **B** grouped by emotion.

The recognition accuracy for all performers was significantly above the chance level (i.e., 12.5%, *p*s < .001; see Fig. 4), and their accuracy varied significantly, which was supported by a one-way repeated-measures ANOVA, *F*(5, 105) = 49.960, *p* < .001, *η*_*p*_^2^ = 0.704. Post hoc comparison with Bonferroni adjustments further showed that Performer 2’s bodily emotion expression was the most recognizable among the performers (*p*s ≤ .001), while Performer 3 had significantly lower recognition accuracy than the others (*p*s < .001). The accuracy of each emotion for all performers is summarized in Fig. 5. For easy comparison, accuracy data are organized in two ways: (A) grouped by performer and (B) grouped by emotion.

The gender distribution among performers was unbalanced, with a male/female ratio of 2:4. To examine the potential effects of gender on emotion recognition accuracy, a two-way ANOVA was applied, with gender as the between-subject factor and emotion as the within-subject factor. Results showed no significant main effect of gender, *F*(1, 4) = 1.052, *p* = 0.363, *η*_*p*_^2^ = 0.208, or interaction effect between gender and emotion, *F*(7, 28) = 0.459, *p* = 0.856, *η*_*p*_^2^ = 0.103, indicating that performers’ gender did not affect how well their emotion expression was identified.

To estimate recognition accuracy for different emotions, we conducted further statistical analysis, which can be found in the following sections.

### Bodily emotion recognition accuracy across different emotions

**Fig. 6:**
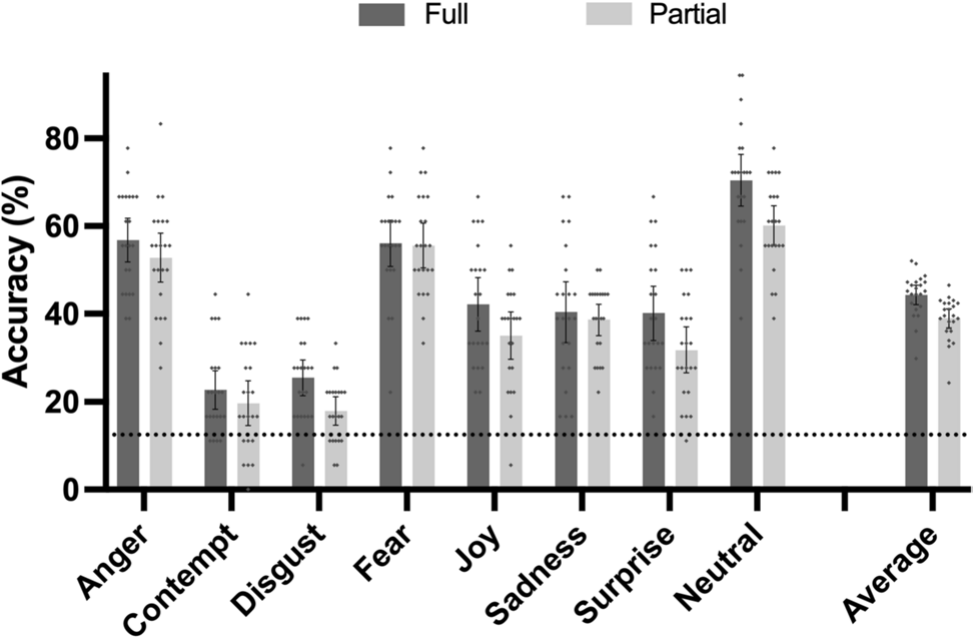
Mean and 95% CI of recognition accuracy for emotions in full and partial marker conditions

The average emotion recognition was 41.6% (*SD* = 4.5%). All the emotion categories were detected significantly above chance (12.5% in an 8-forced-choice task) in both partial and full marker conditions (Fig. 6, one-sample *t*-test results in Supplementary Table 3). This is consistent across all participants (Supplementary Figure 1).

To better understand the effect of additional markers for emotional recognition in bodily movement, we conducted a 2 × 8 repeated-measures ANOVA with the following two factors: emotions (anger/contempt/disgust/fear/joy/sadness/surprise/neutral) and marker conditions (full/partial). Mauchly’s test indicated that the assumption of sphericity had been met, χ^2^(27) = 15.40, *p* = .97.

We observed a significant main effect on emotion, *F*(7, 147) = 63.49, *p* < .001, *η*_*p*_^2^ = .75. The post hoc multiple-comparison tests (with Bonferroni adjustments) between each emotion category are included in Supplementary Table 4. Our results showed that neutral (65.3%), fear (55.8%), and anger (54.8%) were best recognized, with significantly higher accuracy than the other five emotions (contempt, disgust, joy, sadness, surprise). Contempt (21.2%) and disgust (21.7%) were the most poorly recognized, with lower accuracy than all other emotions, while joy (38.6%), sadness (39.5%), and surprise (36.0%) were the mid-level emotions.

### Bodily emotion recognition accuracy across scenarios

In addition, we explored the accuracy of emotion recognition across scenarios (Supplementary Table 2). Given the limited range of scenarios and performers in the current study, conducting a comprehensive statistical analysis to compare bodily expressions across similar contexts is challenging. Consequently, we focused preliminary on descriptive findings. First, while certain emotions were better recognized than others, there was a noticeable variation in accuracy across different scenarios even for the same emotion. For instance, Performer 1's anger scenarios yielded divergent levels of recognition accuracy from 22.7% to 97.7%. That is to say, performer or individual style might not be a dominant factor in this case. Meanwhile, bodily emotional expressions in similar scenarios were not equally recognizable. For instance, the recognizability of joy associated with eating varied markedly across performers (e.g., Performer 1: 40.9%, Performer 2: 95.5%, Performer 5: 4.5%), underscoring how the same scenario can elicit different levels of recognizability depending on the performer. In summary, our adoption of diverse emotion-inducing scenarios has provided a new avenue for future researchers to also consider the contextual effect in body emotional expression and understanding.

### Partial and full marker conditions

The main effect of the marker condition was also significant: The full marker condition on average had significantly higher accuracy (44.3%) than the partial condition (39.0%), *F*(1, 21) = 38.47, *p* < .001, *η*_*p*_^2^ = .65. The interaction effect between emotion and marker condition was not significant, *F*(7, 147) = 1.73, *p* = .11, *η*_*p*_^2^ = .08. To confirm the learning effect without the bias of extremely long or high accuracy of individual participants, we conducted a frequency test based on the proportion of participants with a positive score (i.e., more accurate in full marker condition). Twenty of the 22 participants obtained better performance in the full marker condition (binomial frequency test against 0.5, χ^2^(1) = 14.73, *p* < .001, Supplementary Figure 1), suggesting a significant proportion of participants performing better at the full maker condition. Our data corroborate findings from previous studies (e.g., Dittrich et al., 1996; Pollick et al., 2001) by demonstrating that basic emotions can be identified from point-like biological motion, with average accuracy of 41.6%. It was unexpected, however, that the neutral state was the state that benefited most from having additional markers. The partial marker condition contained less information in the trunk and head. To understand the beneficial effect better, we analyzed the participants’ error trials next.

### Error analysis: Bodily emotion confusion matrix

**Fig. 7:**
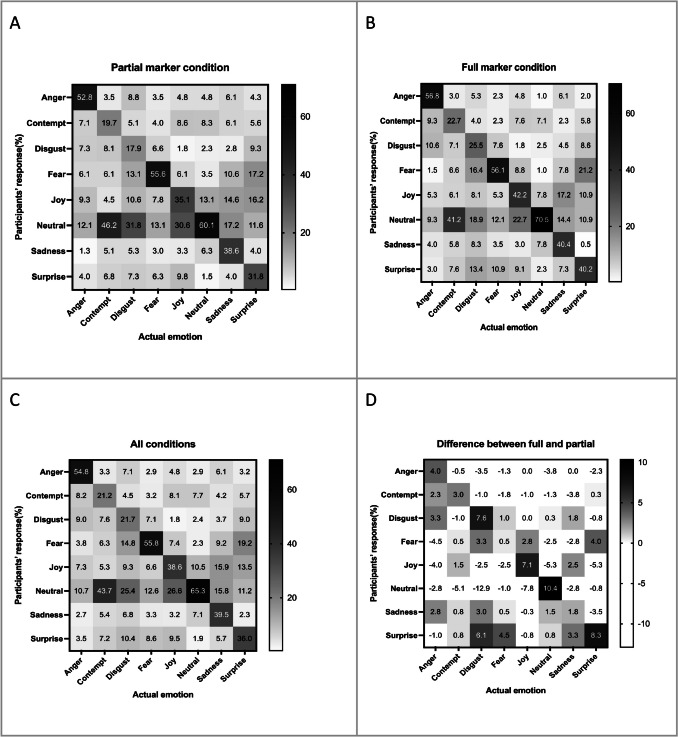
Confusion matrix results for **A** partial marker condition, **B** full marker condition, **C** all conditions combined data, and **D** difference between full marker and partial maker condition (full − partial)

To obtain a more complete picture of how some emotions were mistaken for others, we analyzed the participants’ error responses with a confusion matrix in the partial marker condition (Fig. 7A), full marker condition (Fig. 7B), and all conditions combined (Fig. 7C). We discovered that our forced-choice emotion classification experiment produced results similar to those of past studies (e.g., Dittrich et al., 1996; Atkinson et al., 2004; Volkova et al., 2014b) that used biological motion videos.

Neutral was the best-recognized condition in both partial (60.1%) and full (70.5%) marker conditions, but it was a major confusion option for other emotions, especially for contempt, disgust, and joy. To be exact, 43.7% of contempt, 25.4% of disgust, and 26.6% of joy were mistaken as neutral emotion. Regarding marker set condition, neutral was the one which benefited most (10.4%) from having additional markers, followed by surprise (8.3%) and joy (7.1%). The benefit of additional markers for neutral emotion came from the reduction of false alarms distributed to other emotions, most notably joy (5.3%).

Contempt (21.2%) and disgust (21.7%) had the lowest recognition accuracy, resulting from high inaccurate responses, with a tendency to misjudge the emotions as neutral. On the other hand, disgust was easily confused with fear. Interestingly, the neutral misperception (−12.9%) for disgust was reduced by additional markers (Fig. 7D), which is consistent with the abovementioned observation that neutral emotion benefited from additional markers.

Sadness and surprise were among the mid-range of accuracy. They were both more likely to be misjudged as neutral and joy than other emotions. Surprise was also easily misrecognized as fear, which is easier to understand, as the scenarios frequently seemed to contain a mix of emotions (e.g., surprise party or scared by an earthquake).

## Discussion

### The importance of bodily emotion databases

Through this investigation, we have successfully collected novel data on the Asian body movement of emotional expressions. Professional performers were invited to perform three scenarios for seven basic emotions (joy, anger, sadness, fear, contempt, disgust, surprise) and neutral emotion in a motion-capture laboratory. All the performance scenarios were written by performers based on their past experience, effectively leveraging personalized stimuli to induce desired emotional responses and various situations to increase action diversity. In the subsequent evaluation experiment, we reduced the motion-capture recordings into point-light displays (18 or 57 markers) and invited local participants to judge the perceived emotions. Data analysis revealed that the emotion discrimination accuracy of this experiment is comparable to Western databases that contain fewer performance scenarios. We discovered that certain emotions (anger and fear) were more identifiable from body movement, while others (contempt and disgust) were expressed more subtly.

### Emotion recognition accuracy from body movement

Anger and fear are emotions significantly better recognized than other emotions in the current study, but interestingly, there is little consensus on emotional recognition priority in the research field of body movements with point-light figures. Because point-light figures only contain body motion information, without information on facial emotion, body volume, or skin, it helps us address the question of how motion informs us of emotions. Both “positivity priority” and “negative priority” were reported in past studies. Dittrich et al. (1996) tested the classification of six emotions (fear, anger, grief, joy, surprise, and disgust) with 13 point-light whole-body expressions. Seventy-two participants watched two dancers’ video recordings of the above six emotions and performed the emotion classification task. Results showed that joy and grief were better recognized than other emotions. Atkinson et al. (2004) tested five emotions (disgust, fear, anger, happiness, and sadness) by having 10 actors freely portray each emotion within approximately 6 seconds. The dynamic movements presented by point-light displays were all recognized above a chance level, with happiness the most accurately perceived emotion, followed by sadness, fear, anger, and disgust. All detection accuracy (above 60%) was well above chance (20%), and it was unclear whether there was significant superiority in any of the emotions because such a test was not conducted in the report. The authors mentioned that two factors, i.e., the speed and the degree/size of the motion, might be critical to differentiating between emotions in body expressions. This is in line with Pollick et al. (2001) discovery that in point-light-displayed arm movements, the velocity, acceleration, and jerkiness were associated with perceived emotion activation. Another report (Volkova et al., 2014b) evaluated a database consisting of 11 body expressions (six basic emotions: anger, disgust, fear, joy, sadness, surprise; four extra emotion categories: amusement, pride, relief, shame; and neutral) that uses upper-body stick figures from the MPI Emotion Body Expressions Database for Narrative Scenarios (Volkova et al., 2014a). Performers narrated fairy tale stories while their body movements were recorded. While all emotion recognition accuracy rates were above chance (10%), anger (50.4%) and sadness (45.7%) were the best-recognized emotions, followed by relief (40%) and joy (38.3%). The least recognized emotions were surprise (20.1%) and disgust (28.9%). Volkova and colleagues (2014b) conducted motion analysis and found that motion speed, trajectory peaks, and span of motion (distance between the wrist joints) significantly predicted emotion classification response, indicating that observers rely on this kinematic information to differentiate emotions from biological motion.

The varying results in these studies could have a few explanations. First, the performance formats varied from dance and spontaneous body movements, to movements during storytelling. Dittrich et al. (1996) recruited professional dancers to do a short emotional dance (5 seconds) using stereotypical movement patterns without narrative scenarios. Atkinson et al. (2004) employed 10 performance students to use spontaneous body movements to express the emotions as they saw fit, with only minimal guidance (e.g., to express “disgust” over smell or taste but not over “moral misconduct”). In the MPI database, eight amateur actors chose three fairytale stories to read. After they immersed themselves in the narrator’s role and felt the emotion of the characters, they imagined that they were telling the stories to a child or a group of children. In our study, we invited experienced performers with stage experiences to provide daily scenarios to act. They were informed that they could imagine a partner in the scenario (although they only acted alone) and they could include vocal sounds (although the audio was not recorded). Each performance was typically between 5 and 10 seconds. These variations in the formats inevitably created considerable differences. For example, storytellers who sat on chairs only had upper body information to express their emotions. Secondly, the performance scenarios varied in these studies. Dittrich et al. (1996) and Atkinson et al. (2004) did not explicitly ask the performers to act upon a specific scenario, while in our study, each recording came from a life situation that the performer prepared in advance. Volkova et al. (2014a) used fairy tales as materials. This made some emotions (e.g., neutral) appear more frequent than others (e.g., shame). It is also possible that one sentence includes several emotions, and the performers may have to switch emotions within a short amount of time (e.g., the average duration of each video included 6.84 word tokens). Finally, all the abovementioned studies selected different emotion categories, and some emotions may be harder to discriminate (e.g., contempt) than others. When such an emotion is included in the response list, it inevitably alters the confidence level in participants’ responses. One possible way to reconcile the discrepancies of the abovementioned studies is to take the approach of interpretive motion (e.g. Wallbott, 1998) to construct a list of actions for cross-study comparison. This method is ideal but labor-intensive and requires extensive training to reach expertise. An automated motion annotation system would help make this process more feasible and affordable.

Contempt (21.2%) and disgust (23.7%) are the two emotions with the lowest accuracy in the current study, a finding consistent with past studies (Dittrich et al., 1996; Atkinson et al., 2004; Volkova et al., 2014b). This may be because these emotions are more subtle, as they are classified with low arousal and involve fewer body movements. Some scenarios in the categories of contempt (e.g., “I witnessed someone running a red light”; “I sat near someone with disgusting eating habits”) and disgust (e.g., “I encountered a person who had poor hygiene”; “I saw a dirty room”) coincide with neutral scenarios (e.g., walking, standing on the train, daydreaming) in the current study. Additionally, contempt and disgust are expressed more with facial expressions and less with body movements. In Asian culture, contempt and disgust are sometimes held within or concealed, as they concern moral judgment (Tsai, 2007; Soto et al., 2016; Song et al., 2024) and might be less desirable in an environment that strongly values social harmony (Tsai et al., 2002; Schouten et al., 2020). Interestingly, although some performers reported in the interview that contempt and disgust were similar and, therefore, difficult to differentiate during the performance, these two emotions were not highly confusing for the raters who watched the point-light movements.

The confusion matrix results imply that emotional expression is inherently complex and multifaceted. Other studies on emotion recognition for bodily expression also showed confusion between different emotions (Dael et al., 2012; Coulson, 2004; Fourati & Pelachaud, 2014; Fourati & Pelachaud, 2016; Gross et al., 2010; Volkova et al., 2014b). This complexity could be associated with contextual triggers. Certain contexts can trigger mixed emotions, which causes the expression to exhibit characteristics of multiple emotions. For example, in our study, the scenarios for surprise could be surprise with joy (e.g., surprise party) or surprise with fear (e.g., an earthquake suddenly occurred). Results supported the tendency for surprise to be often confused with fear (19.2%) and joy (13.5%). Evidence has shown that participants can recognize the affective state even when the face and body are masked, which addresses the crucial role of context in emotion perception (Chen & Whitney, 2019; Martinez, 2019).

In addition, there are shared body expressions by different emotions. For example, Parkinson and colleagues (2017) found that anger and disgust both involve forceful movements such as arms thrusting downward, while anger and fear share irregular and erratic movement. Coulson (2004) reported that surprise and happiness share similar postures and are confused with each other. The shared body expression is a common challenge in the field of emotion recognition, as also evidenced by studies on facial expressions, where fear and surprise are often miscategorized due to shared facial features (Ekman, 1992). Moreover, emotions of lower arousal levels are prone to misclassification. Wallbott (1998) noted that body movements for certain emotions (despair, terror, pride) might be less distinctive than more intense emotions (e.g., elated joy, hot anger). The role of emotional intensity and its tangled relationship between emotion expressions makes emotion recognition challenging for both human and machine learning systems. Future research is needed on identifying the shared and distinct features in bodily emotional expression.

The benefit of the full marker condition over the partial marker condition may be due to the additional information from the head and torso. For example, the full condition had four markers for the head, whereas the partial condition had only one. This allowed for a clearer depiction of head movements. Additional markers at key joints (wrists, spine, etc.) may convey crucial kinematic information. De Meijer (1989) found that specific body movements, such as variations in speed, amplitude, and directness, play a crucial role in how emotions are perceived. The enhanced visibility and interpretation of these subtle body movements due to the full marker condition could explain the observed advantages.

### Self-created scenarios and cultural implications

Preliminary scenario analysis showed that accuracy varied across both performers and scenarios, which underscores the complexity of emotion recognition and the significant role of context and individual expression styles. Although our current dataset limits the extent of statistical analysis, the apparent variations we have documented serve as a valuable foundation for future research. As we continue to expand our collection of data, including a broader range of performers and scenarios, we anticipate being able to conduct more comprehensive analyses to explore these variations in greater depth.

Most well-known bodily emotion databases utilize predetermined scenarios for the encoding stage. For example, actors who contributed to the GEMEP database were given detailed descriptions of life situations considered “prototypical,” designed specifically to “help the use of acting techniques” (Bänziger et al., 2006). Similarly, scripts were provided to the participants whose motions were captured for the MPI Emotional Body Expressions Database for Narrative Scenarios (Volkova et al., 2014a), the Emotional body expression in daily actions database (Fourati & Pelachaud, 2014), and the motion-capture library for the study of identity, gender, and emotion perception from biological motion (Ma et al., 2006), to name a few.

While the professional actors in this experiment were well qualified to perform scenarios presented to them, we were curious about the insight their unique contributions could bring; thus we opted for a different design in this experiment and asked the participants to create their own prompts. Indeed, during data processing, we discovered cultural nuances which added a layer of complexity to the translation from Japanese to English. Although several scenarios seemed perplexing at first, upon further investigation, we were able to glean insight into the influence of culture and individual differences on emotions.

In the neutral category, a Japanese performer created the following script: “上司に怒られた (My boss got angry with me).” Initially, the performer’s categorization seemed puzzling, as the situation appears more closely associated with fear, anger, or even sadness. From a later interview, however, we learned from the performer that in the Japanese context, where work culture is dominated by a rigid hierarchy, being the recipient of a supervisor’s wrath is considered a common occurrence. People have therefore learned to be indifferent in such situations. To contextualize the translation, we added an extra detail that resulted in “I zoned out when my boss got angry with me.”

A number of scenarios from the disgust category share the universal theme of occurrences that offend the five physical senses; our actors wrote about seeing, tasting, hearing, touching, and smelling something unpleasant. However, the specific triggers may vary depending on both cultural and individual factors. We observed a surprising anomaly in the following scenario: “The train's departure has been delayed endlessly due to an accident.” This was not categorized under anger, as we might expect. The emphasis on an accident could potentially also evoke sadness in some people, but the prioritization of the endless delays seems more reasonable for anger. Instead, this situation was perceived with disgust, as the actor focused on condemning the train’s lack of punctuality as well as the railroad organization’s ostensible inefficiency in dealing with the accident. In the Japanese context, punctuality and efficiency are both highly valued attributes, and thus their deficiency evokes disgust.

Another scenario from this category caught our attention: “I want to leave after finishing drinks at the first bar, but I'm being guilt-tripped into joining the after-party.” To contextualize this situation, Japanese employees are frequently expected to join after-work social gatherings dominated by drinking alcohol together. Rejecting such an invitation is essentially a social faux pas. In this case, the actor experiences social pressure as a form of offense, and thus disgust.

## Limitations and future directions

In the current study, we took a novel approach to create an Asian-based database for bodily emotion expressions, integrating diverse scenarios to produce reliably recognizable emotions. Our results are promising despite the limitations in (1) emotion categories (currently we have seven basic emotions and neutral only), (2) number of performers (currently we have six trained professional performers), (3) the evaluation test (currently we ask emotion categorization), and (4) the gender imbalance among raters in the evaluation test. Building on the initial findings, our future research will continue to complete the database and pursue further aspects as follows:

First, the current study can be extended to categories beyond basic emotions such as social emotions that occur during interpersonal interaction (e.g., gratitude, jealousy, pride, guilt). Second, we plan to recruit a broader spectrum of performers varied in personification information (e.g., gender, age, performance experience), and include more raters in the evaluation experiment. Third, to cover the complexity of emotion recognition, future research needs to implement tasks that assess emotion intensity or in other dimensions (e.g., valence, arousal, sociality), which will enable a more nuanced understanding of emotional expressions. This includes developing sophisticated methodologies for annotating performances with multiple emotion labels and their respective intensity level. Moving forward, our database will include objective metrics (accuracy, intensity, ambiguity, etc.) for each performance. This will provide researchers with a diverse set of stimuli, each with varying levels of metrics, thereby enriching the potential for nuanced research and application. In addition, our study utilized self-prepared scenarios for the first time, inviting performers' personalized experiences to be included. This opens an additional avenue through which to investigate the relationship between contextual cues and bodily emotion that was previously impossible. The rich semantic information derived from these scenarios can be helpful for machine learning or other types of analyses. Finally, an Asian-based body emotion database will empower the pursuit of understanding cross-cultural differences in a more systematic way. Studies have pointed out that humans use universal gestures (e.g., Witkower et al., 2021; Fay et al., 2022; Muratova et al., 2021), although the local culture has been shown to modulate the expressions as well (e.g., de Gelder & Huis in 't Veld, 2016; Lim, 2016). While the current bodily emotion recognition test cohort includes different nationalities, all participants are from East Asia. It will be interesting to examine whether the proximity of cultures could also lead to the sharing of tacit knowledge related to body language, which in turn could allow for easier emotion recognition (e.g., in-group advantage, Elfenbein & Ambady, 2003; Matsumoto et al., 2009; Elfenbein, 2013; Parkinson et al., 2017). Recruiting participants of other ethnicities and cross-database comparison will be instrumental in shedding light on the extent of cultural influence in this process (e.g., Marsh et al., 2003; Beaupré & Hess, 2006).

## Conclusion

We applied an innovative emotion stimulation approach (personalized scenarios) to develop the first Asian body movement database of emotion with a point-light display. Professional performers provided individual performance scenarios that are personally relevant to whole-body movements. Our results are promising and encouraging, and provide the first step toward establishing a database for underrepresented groups in our research community.

## Supplementary information

Below is the link to the electronic supplementary material.

Supplementary file1 (DOCX 3675 KB)

## Data Availability

The data and analysis code are available for other researchers via: https://osf.io/ta9np/

## Notes

Availability of code

The analysis code is available for other researchers via: https://osf.io/ta9np/
