# Supplementary material for: Toward an Asian-based bodily movement database for emotional communication
Source: Behav Res Methods. 2024 Dec 10;57(1):10. doi: 10.3758/s13428-024-02558-2 (PMC11632091; doi:10.3758/s13428-024-02558-2)
Supplement: Supplementary file 1 — Supplementary file1 (DOCX 3675 KB) [file 13428_2024_2558_MOESM1_ESM.docx]

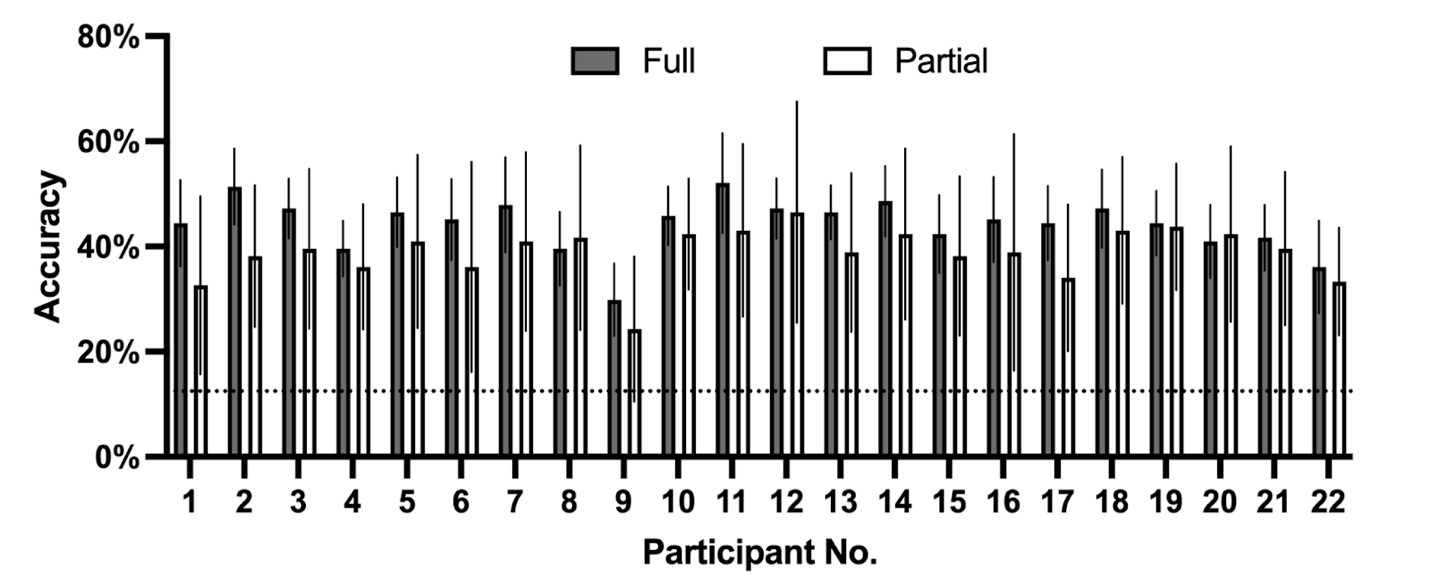


Supplementary Figure 1   Mean and 95%CI of recognition accuracy of emotions in full and partial markers conditions for individual participants (N=22).

**Supplementary Table 1.** The emotion definition list in English and Japanese

| Emotion | Definition |
| --- | --- |
| Joy | Experiencing an extraordinary feeling of pleasantness, well-being and sensual delight 特別な心地よさ、幸福感、官能的な喜びを体験できる |
| Sadness | Feeling discouraged by the irrevocable loss of a person, thing, or place 人、物、場所などの取り返しのつかない損失に落胆すること |
| Anger | Extreme displeasure caused by someone’s stupid or hostile action 誰かの愚かな行動や敵対的な行動によって引き起こされる極度の不快感 |
| Surprise | An emotion triggered by abruptly facing an unexpected and unusual event (without positive or negative connotation 予期せぬ異常な出来事に突然直面すること（ポジティブな意味合いもネガティブな意味合いもない） |
| Fear | An intense feeling experienced when facing imminent danger that threatens our survival or physical well-being 生存または身体の健康を脅かす差し迫った危険に直面すること。 |
| Disgust | Revulsion when faced with an unpleasant object or environment 不快な物体や環境に直面したときの反発。 |
| Contempt | Disapproval of the socially or morally reprehensible conduct of another person 他人の社会的または道徳的に非難されるべき行為に対する不支持 |
| Neutral | A feeling of indifference 感情を出さない。 |

**Supplementary Table 2.** The performance scenario list prepared by 6 professional performers. The recognition accuracy from 22 participants was averaged across partial and full marker conditions.

|  | **Scenario list from performer 1-6 (3 per performer)** | **Recognition Accuracy** |
| --- | --- | --- |
| **Joy** |  | **38.6%** |
| **1** | I realize that it is my day off | 29.5% |
|  | I am eating my favorite ramen for the first time in half a year | 40.9% |
|  | I see that the sky is filled with stars | 25.0% |
| **2** | I opened the curtains and the sky was beautiful | 27.3% |
|  | I am eating grilled meat | 95.5% |
|  | The flowers in my house are blooming | 50.0% |
| **3** | I made it to the finals of a dance competition | 68.2% |
|  | I passed an exam | 11.4% |
|  | The person I like confesses his/her love to me | 13.6% |
| **4** | A grandma is watching the video stream (Zoom) of her grandson walking for the first time. | 54.5% |
|  | I am under the cherry blossom trees in the park when the flowers are in full bloom. | 79.5% |
|  | I am sitting in the front passenger seat of a car driven by my lover. | 9.1% |
| **5** | When I'm having a meal | 4.5% |
|  | Reunion with friends | 90.9% |
|  | Winning a lottery ticket | 31.8% |
| **6** | When I am told that I can eat as much konamon as I like  (Note: Konamon, 粉もん, meaning "flour things" in Japanese, refers to a variety of savory dishes made with wheat flour in the Kansai region of Japan.) | 43.2% |
|  | When my boyfriend told me that I looked cute today and patted my head | 11.4% |
|  | When I am complimented by my favorite teacher whom I respect | 9.1% |
| **Sadness** |  | **39.5%** |
| **1** | I broke up with my girlfriend | 29.5% |
|  | I cannot afford to miss this train, but the train doors close before I can make it inside. | 2.3% |
|  | My employee makes the same mistake over and over again | 54.5% |
| **2** | I lost my earrings | 97.7% |
|  | My girlfriend broke up with me | 34.1% |
|  | My cat died | 75.0% |
| **3** | My grandmother passed away | 70.5% |
|  | The watch that I treasure is broken | 4.5% |
|  | I couldn't get a ticket for the show I wanted to go to | 52.3% |
| **4** | I ruminated about my father's death in the middle of the night. | 88.6% |
|  | HR informed me that my work contract would not be renewed. | 11.4% |
|  | I dropped the tableware I cherished and broke it. | 31.8% |
| **5** | Death of relatives | 2.3% |
|  | Farewell to friends | 38.6% |
|  | Breaking up | 56.8% |
| **6** | When my parents died | 31.8% |
|  | When I lost a matching ring and couldn't find it | 6.8% |
|  | When my parents' house was burned down by a fire | 22.7% |
| **Anger** |  | **54.8%** |
| **1** | I received an unreasonable request | 22.7% |
|  | An important appointment is canceled | 86.4% |
|  | Something I cherish is broken | 97.7% |
| **2** | Someone bumped into me but didn't apologize. | 81.8% |
|  | Someone said unreasonable things to me | 97.7% |
|  | Someone broke my stuff. | 81.8% |
| **3** | I got angry about something unreasonable. | 15.9% |
|  | A passerby bumped into me but did not apologize. | 43.2% |
|  | Being forcibly overtaken by a car | 9.1% |
| **4** | Having a disagreement with my lover while dining at a restaurant. | 72.7% |
|  | I am telling my friend that my company is corrupt and takes advantage of me. | 38.6% |
|  | Watching TV news of Russia's invasion of Ukraine. | 54.5% |
| **5** | People dying in a war | 95.5% |
|  | Having my effort ridiculed | 25.0% |
|  | Someone secretly ate my favorite food I had wanted to save for later. | 4.5% |
| **6** | When I was called a drama queen | 90.9% |
|  | When my opinion is completely disregarded | 27.3% |
|  | When people make snarky remarks like "Such a beginner/What a beginner." | 40.9% |
| **Surprise** |  | **36.0%** |
| **1** | A cicada suddenly flies toward me. | 27.3% |
|  | An earthquake suddenly occurred | 20.5% |
|  | Oil splashes while cooking | 45.5% |
| **2** | I heard a loud noise | 13.6% |
|  | I ran into a friend | 15.9% |
|  | Someone was urgently calling my name | 52.3% |
| **3** | A loud noise came out of nowhere | 47.7% |
|  | The floor was flooded | 11.4% |
|  | Water fell on my neck | 9.1% |
| **4** | Learning that I have won a bicycle when I saw my lottery numbers in the papers. | 45.5% |
|  | I was on a bus, the bus suddenly braked, and had a minor rear-end collision. | 54.5% |
|  | At Tokyo Station, I ran into a friend who was supposed to be abroad. | 56.8% |
| **5** | Surprise party | 84.1% |
|  | Bugs appearing | 22.7% |
|  | An earthquake occurring | 15.9% |
| **6** | When I woke up in the morning and became an animal | 75.0% |
|  | A giant stood in front of me when I was walking | 4.5% |
|  | When I suddenly fall into a pit | 45.5% |
| **Fear** |  | **55.8%** |
| **1** | I heard a noise at a haunted place | 88.6% |
|  | I am surrounded by several gangsters | 88.6% |
|  | I encountered a bear in the forest | 11.4% |
| **2** | I am crossing a suspension bridge | 70.5% |
|  | I felt being chased | 90.9% |
|  | I felt a bug on my skin | 47.7% |
| **3** | I got sick, and I may lose my voice as a result. | 4.5% |
|  | I looked down from a high place | 0.0% |
|  | I almost got hit | 61.4% |
| **4** | I'm on an airplane and the turbulence is unusually intense. | 9.1% |
|  | I was the first to get my jab at the vaccination site. | 95.5% |
|  | During a stay at the hotel, the fire alarm went off, and I could smell the smoke. | 70.5% |
| **5** | Moments before taking a leap in bungee jumping | 84.1% |
|  | When I met a bear | 59.1% |
|  | Being driven around by a novice driver. | 52.3% |
| **6** | When faced with a murderer | 52.3% |
|  | When surrounded by zombies | 50.0% |
|  | When the whole body was covered in blood | 68.2% |
| **Contempt** |  | **21.2%** |
| **1** | I witnessed someone running a red light | 6.8% |
|  | I sat near someone with disgusting eating habits | 11.4% |
|  | I am repeatedly being told a boring story | 15.9% |
| **2** | I saw someone littering a cigarette butt | 56.8% |
|  | I was bad-mouthed by a friend | 63.6% |
|  | I see someone annoying another person | 77.3% |
| **3** | I saw a person who did not improve no matter how many times I warned him or her | 13.6% |
|  | I saw someone sweating like a waterfall but didn't do anything about it. | 13.6% |
|  | I smelled a strange odor | 15.9% |
| **4** | There is an unmasked person coughing all the time in the cafe. | 15.9% |
|  | I met a man who exposed himself to me while walking about the town. | 0.0% |
|  | Drinking with a man who is always bragging about himself. | 2.3% |
| **5** | People scattering their luggage around the train and blocking the way. | 38.6% |
|  | When the other party is late | 6.8% |
|  | When I saw a false accusation | 4.5% |
| **6** | When I meet someone who only badmouths others | 15.9% |
|  | When talking to someone who is condescending in every way. | 4.5% |
|  | When you meet someone who treats everyone differently depending on their status. | 18.2% |
| **Disgust** |  | **21.7%** |
| **1** | I am trying to get rid of a fly | 47.7% |
|  | I ate ice cream during a diet | 65.9% |
|  | I encountered a person who had poor hygiene | 56.8% |
| **2** | I touched something dirty | 2.3% |
|  | I am shown grotesque images | 61.4% |
|  | The music is too loud. | 2.3% |
| **3** | I see a crowd of people | 0.0% |
|  | I ate food I didn't like | 0.0% |
|  | I found a disgusting insect | 15.9% |
| **4** | A giant bug came into the room. | 4.5% |
|  | I want to leave after finishing drinks at the first bar, but I'm being guilt-tripped to join the after-party. | 25.0% |
|  | The train has been delayed due to an accident and has been waiting at the platform endlessly. | 4.5% |
| **5** | When I smell bad odors | 15.9% |
|  | I touched a slimy thing | 9.1% |
|  | I saw a dirty room | 0.0% |
| **6** | When a bird flies in front of you | 72.7% |
|  | When I bump into someone with an unpleasant history | 4.5% |
|  | When surrounded by a large number of bugs | 2.3% |
| **Neutral** |  | 65.3% |
| **1** | I was on the train | 75.0% |
|  | I watch the clouds drift by. | 72.7% |
|  | I am drinking coffee | 93.2% |
| **2** | I zoned out when my boss got angry with me  (Note: The original scenario was “My boss got angry with me” (“上司に怒られた”). The performer explained that in the Japanese context where work culture is dominated by a rigid hierarchy, being the recipient of a supervisor’s wrath is considered a common occurrence. People have therefore learned to be indifferent in such situations. To contextualize the translation, we added an extra detail that resulted in “I zoned out when my boss got angry with me.” ) | 31.8% |
|  | I lost my belongings | 20.5% |
|  | I am eating | 65.9% |
| **3** | When I just woke up in the morning | 77.3% |
|  | Listening to a story that doesn't interest me | 4.5% |
|  | When I'm zoning out at home | 36.4% |
| **4** | Listening to the seminar and taking notes. | 81.8% |
|  | Giving instructions to the assistant regarding the day's agenda. | 84.1% |
|  | Ordering coffee at a cafe. | 93.2% |
| **5** | Walking around in a daze | 70.5% |
|  | When I am tired | 36.4% |
|  | When listening to a long story | 93.2% |
| **6** | When putting on makeup | 72.7% |
|  | When scrolling through a smartphone | 79.5% |
|  | When I am in the bathroom | 86.4% |

**Supplementary Table 3.**

**All emotional recognition accuracies are significantly above the chance level (12.5%).**

| **Emotions** | **Full Marker**  **t (df=21)** | ***p*** | **Partial Marker**  **t (df=21)** | ***p*** |
| --- | --- | --- | --- | --- |
| Anger | 18.504 | < .001 | 15.030 | < .001 |
| Contempt | 4.805 | < .001 | 2.944 | .004 |
| Disgust | 6.646 | < .001 | 4.942 | < .001 |
| Fear | 17.217 | < .001 | 17.559 | < .001 |
| Joy | 10.174 | < .001 | 8.798 | < .001 |
| Sadness | 8.421 | < .001 | 15.429 | < .001 |
| Surprise | 9.388 | < .001 | 7.777 | < .001 |
| Neutral | 20.403 | < .001 | 19.365 | < .001 |
